# Linear and exponential sunscreen behaviours as an explanation for observed discrepancies in sun protection factor testing

**DOI:** 10.1111/phpp.12500

**Published:** 2019-09-05

**Authors:** Carles Trullàs, Corinne Granger, Henry W. Lim, Jean Krutmann, Philippe Masson

**Affiliations:** ^1^ Innovation and Development ISDIN Barcelona Spain; ^2^ Department of Dermatology Henry Ford Hospital Detroit MI USA; ^3^ IUF – Leibniz Research Institute for Environmental Medicine Düsseldorf Germany; ^4^ Phi consulting Bordeaux France

**Keywords:** exponential, indoor, linear, outdoor clinical, sun protection factor

## Abstract

**Background:**

In vivo testing of sun protection factor (SPF) values can show considerable interlaboratory variability. We studied the underlying reasons and clinical implications.

**Methods:**

Following the ISO 24444:2010 SPF testing method, seven contract research organizations (CROs) tested eight sunscreens marketed as SPF50 or SPF50+ and the reference SPF15 sunscreens P2 and P3 and SPF43 P6. We analysed differences in the products and CRO testing methods with regard to SPF variability. We tested the erythema prevention capacity of five of the products in subjects exposed to high doses of natural sunlight in Mauritius.

**Results:**

Sun protection factor values varied dramatically between different CROs for some, but not all of the sunscreens. Those with the largest variability had an SPF50+, and their SPF values differed from a maximum of 62.4 to a minimum of 5.5. These products did not share a common sun‐filter composition, and some CROs used low and others high irradiation dose regimens. When comparing these two regimens, test products fell into two categories: (i) they either behaved similarly (“linear”) or (ii) they behaved differently (“exponential”). In the outdoor clinical study, exponential and linear sunscreens did not differ in their photoprotection capacities.

**Conclusion:**

Differences in reported SPF values depend on the linear vs exponential behaviour of such products if subjected to low‐ vs high‐dose test regimens. Under real‐time exposure to natural sunlight, exponential and linear sunscreens did not differ in their erythema prevention capacity. Laboratory SPF testing of exponential sunscreens bears the risk of underestimating their in‐use SPF.

## 1 INTRODUCTION

Forty years after the publication of the first in vivo sun protection factor (SPF) laboratory evaluation method (FDA 1978),^1^ and despite the existence of two methods considered the gold standard in their respective applicable areas (the ISO 24444:2010^2^ and FDA 2011^3^ methods), the large inter‐laboratory variability in SPF testing values is still a challenge. A recent study found that the variability in the SPF factors obtained by different clinical research organizations (CROs) showed an increase parallel to the enlargement of the expected protection factor.^4^ To date, attempts to find an explanation for these differences have mainly taken into account factors such as equipment, operator, process, environment and management in the testing methods.^4^, ^5^ An alternative explanation might be, however, that even when the same method is applied, different sunscreens behave differently. This assumption is supported by the observation of Damian et al, who reported that different sunscreens behaved differently when subjected to standardized SPF testing.^6^ Specifically, they found that the SPF of sunscreens is dependent on MED (minimal erythema doses) and that there was a reverse correlation between the MED of unprotected skin and the resulting SPF value with the same product.

In this study, we aimed to build on this observation and assess its clinical relevance. To this end, we selected 8 commercially available products claiming SPF50/SPF50+ and the two reference standard sunscreens SPF15 (P2 and P3) and the reference standard sunscreen SPF43 (P6) of the ISO 24444:2010 and ISO/DIS 24444:2019, respectively. We used several CROs to evaluate their SPF values according to the in vivo SPF laboratory method ISO 24444:2010.^2^ In addition, for 5 of the products, we evaluated their capacity to prevent erythema at 24 hours after exposure in outdoor conditions under very high or even extreme exposure to natural sunlight.

## 2 MATERIALS AND METHODS

### 2.1 Materials

We collected samples of eight marketed sunscreens (S1 to S8) claiming SPF50 or SPF50+. These sunscreens had different textures and compositions and were manufactured by different companies; we also included samples of the ISO 24444:2010 and ISO/DIS 24444:2019 reference standards, P2, P3 and P6 (Table 1).

**Table 1:** Qualitative composition of filters in the marketed sunscreens and P2, P3 and P6 reference standards

Filter	S1	S2	S3	S4	S5	S6	S7	S8	P2	P3	P6
Butyl methoxydibenzoylmethane	X	X	X			X	X	X		X	
Ethylhexyl methoxycinnamate	X			X	X		X			X	X
Ethylhexyl triazone	X	X		X	X	X	X				
Bis‐ethylhexyloxyphenol methoxyphenyl triazine		X	X	X		X		X			X
Titanium dioxide	X		X				X				
Octocrylene		X	X			X		X			
Diethylamino hydroxybenzoyl hexyl benzoate				X	X						
Phenylbenzimidazole sulfonic acid								X		X	
Homosalate						X	X				
Ethylhexyl salicylate		X				X					
Ethylhexyl dimethyl PABA									X		
Benzophenone‐3									X		
Methylene bis‐benzotriazolyl tetramethylbutylphenol								X			X
Terephthalylidene dicamphor sulfonic acid		X									
Drometrizole trisiloxane						X					

### 2.2 Methods

#### 2.2.1 In vivo indoor SPF testing studies

The SPF of eight marketed sunscreen formulations (S1 to S8) and the reference standards P2, P3 and P6 were evaluated in vivo by seven CROs in accordance with the International Standard ISO 24444:2010 SPF Test Method.^2^ This laboratory method determines the protection provided by sunscreen products on human skin against erythema induced by ultraviolet radiation emitted by a solar simulator. All SPF testing was performed in line with Good Clinical Practice principles and the requirements of the Declaration of Helsinki. Test subjects were informed about the study, and informed consent was obtained from each subject.

#### 2.2.2 Outdoor clinical study

In an intraindividual, single‐centre, double‐blind, randomized clinical study, approved by an independent Ethics Committee, located at King George V Corner, Floréal, Mauritius, we compared the erythema prevention capacity of the investigational products S1 and S7 against P3 and comparator sunscreens S2 and S3, after very high and extreme exposure to natural sunlight. The test was performed on the island of Mauritius, during summertime in Tamarin City (latitude −20°19' 32.02"S/ longitude 57°22' 14.02"E, altitude 460 m above sea level). The intensity of UV radiation was calculated according to time and day of exposure by the department of applied physics of the University of Barcelona. Thirty‐five healthy volunteers (skin phototype II to IV) were recruited.^7^ The main evaluation criterion was the erythema score at 24 hours after the end of sunlight exposure on 6‐grade scale from 0 (no erythema) to 5 (very severe erythema with blistering).^8^ The tested sunscreens were applied at 2 mg/cm^2^ on selected areas of the volunteers’ backs. At baseline, the erythema score was 0. The subjects were asked to lie on their front while being exposed to the sun for 2 hours; the non‐sunscreen‐protected area was covered with fabric after 1 hour of exposure in all subjects.

To statistically analyse the differences in erythema prevention by the test products, a one‐way ANOVA was done with *products* as the fixed factor and *subject* as random components, followed by Tukey's post hoc procedure for pairwise comparison between means. The analysis was performed on rank‐transformed data since the abnormality assumption of the standardized residuals was violated at 1% level of Shapiro‐Wilk test.

## 3 RESULTS AND DISCUSSION

### 3.1 In vivo indoor SPF testing studies

To corroborate and expand on the observation by Damian et al,^6^ we first asked a total of 7 CROs to test 8 different marketed sunscreens and the reference standard sunscreens P2, P3 and P6. As can be seen from Table 2, SPF test results varied substantially between different CROs for some of the test products. These differences were particularly striking for sunscreens with an SPF50 or SPF50+.

**Table 2:** SPF values as measured by several CROs (a‐g), all products

Product	Labelled SPF	Tested SPF	CRO
S1	50+	60.4	a
		62.4	b
		51.3	c
		15.8	d
		5.5	e
		10.2	e
S2	50+	43.8	f
S3	50+	60.1	b
		61.0	e
S4	50+	64.5	a
		21.1	d
		17.5	e
S5	50	59.5	a
		6.2	d
		5.3	e
S6	50+	65.6	f
S7	50	66.3	a
		53.0	c
		8.6	d
		9.8	e
S8	50+	66.0	a
		60.1	b
		61.9	e
P2	16.1	15.4	a
		15.9	d
		16.8	e
P3	15.7	16.1	b
		15.2	g
		15.0	f
P6	43	51.8	c
		51.5	g
		53.4	f

Abbreviations: CRO, contract research organization; SPF, sun protection factor.

We next wondered if these differences might be due to a specific ultraviolet (UV) filter combination or galenic formulation. As can be seen from Table 1, the sunscreens that showed the largest inter‐laboratory SPF variability (S1, S4, S5 and S7) did not share a common sun‐filter composition, but they did share the galenic form of a water‐rich gel. It is therefore unlikely that the observed differences in SPF testing can be attributed to a specific sun‐filter combination, but we cannot rule out other factors such as differences in UV filter concentrations.

We next looked in detail at the ISO 24444:2010 SPF test method itself and the way the CROs had determined the SPF. Interestingly, we found that some CROs were using low dose irradiation (D1), and others were using higher irradiation dose regimens (D2). These different UV dose regimens applied by CROs are likely due to their previous training, education and skills in reading unprotected MED.

We next used this classification (high dose vs low dose) to categorize differences in SPF testing for the same sunscreen products. As a consequence, we were able to classify sunscreens into 2 groups: (i) products that obtained the same level of SPF values and were not affected by the radiation dose delivered and (ii) products that had different levels of SPF depending on the radiation dose delivered.

We therefore subdivided the tested products into one group which showed a linear behaviour (the SPF varied only slightly, depending on the level of irradiation used by the CROs) and one group which showed an exponential behaviour (the SPF varied clearly depending on the level of irradiation used by the CROs) (Table 3). According to this definition, we classified the sunscreens S2, S3, S6, S8, P2, P3 and P6 as linear sunscreens, while the sunscreens S1, S4, S5 and S7 were classified as exponential sunscreens with SPF values that varied from 5.3 to 59.5 for the same product (Table 3).

**Table 3:** Sun protection factor (SPF) as measured by different CROs, divided into low and high irradiation doses and categorization of the products as linear (L) or exponential (E)

Product	Category	Labelled SPF	SPF results D1			SPF results D2		
			D1 (mJ/cm^2^)	SPF	CRO	D2 (mJ/cm^2^)	SPF	CRO
S1^a^	E	50+	5.7	60.4	a	21.5	15.8	d
			4.7	62.4	b	37.8	5.5	e
			4.3	51.3	c	38.7	10.2	e
S2^a^	L	50+	–	–	–	17.3	43.8	f
S3^a^	L	50+	4.6	60.1	b	34.5	61.0	e
S4	E	50+	5.7	64.5	a	22.3	21.1	d
						35.5	17.5	e
S5	E	50	5.7	59.5	a	22.0	6.2	d
						35.4	5.3	e
S6	L	50+	–	–	–	18.5	65.6	f
S7^a^	E	50	6.0	66.3	a	22.6	8.6	d
			4.7	53.0	c	45.0	9.8	e
S8	L	50+	6.4	66.0	a	34.1	61.9	e
			4.0	60.1	b			
P2	L	16.1	5.1	15.4	a	22.3	15.9	d
						35.4	16.8	e
P3^a^	L	15.7	4.6	16.1	b	19.8	15.2	g
						20.4	15.0	f
P6	L	43	4.7	51.8	c	22.3	51.5	g
						17.3	53.4	f

Abbreviations: CRO, contract research organization; D1, irradiated dose (low range); D2, irradiated dose (high range); E, exponential; L, linear.

a Products also tested in outdoor conditions.

**Figure 1:**
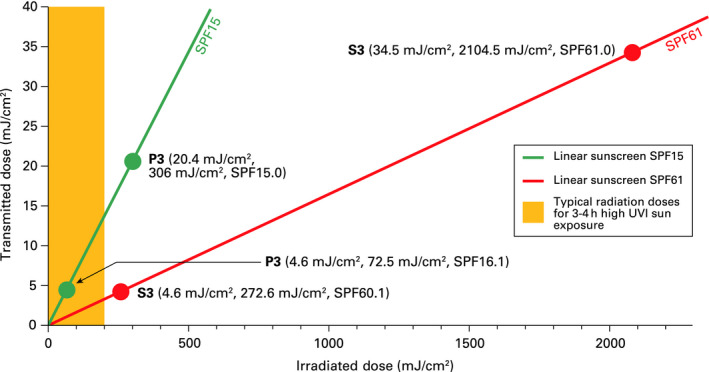
Illustration showing the linear behaviour of sunscreens and the data for S3 and P3 (Table 3)

Figure 1 displays the transmitted vs irradiated dose included in Table 3 for sunscreens S3 (red dots) and P3 (green dots). It can be observed that at both low‐ and high‐dose ranges the SPF was constant (SPF is the inverse of the slope of the lines). The orange shaded area corresponds to the typical sun exposure doses transmitted during 3‐4 hours of exposure under a high UV index (UVI). It should be mentioned that in the evaluation of S3, the protected skin was exposed to a dose of more than 2000 mJ/cm^2^, which was more than 9 times the dose received in real sun exposure in a high‐intensity UVI setting (less than 260 mJ/cm^2^). In the in vivo SPF laboratory tests, it is assumed that the use of these high doses to evaluate the SPF should not modify the behaviour of the sunscreen.

**Figure 2:**
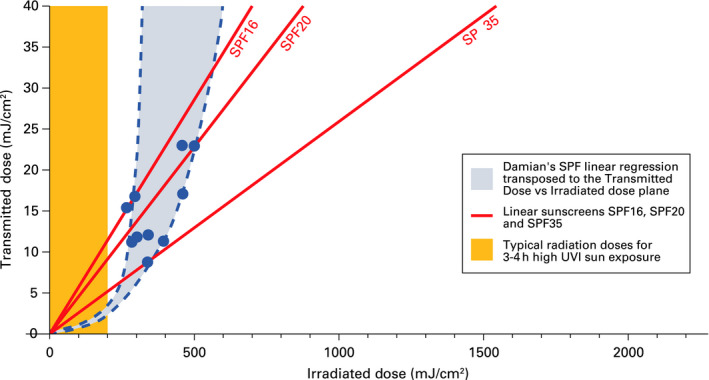
Illustration showing the exponential behaviour of sunscreen based on data from Damian et al^6^

As has been previously pointed out, Damian et al^6^ showed that SPF was not a constant and independent factor from the MED and that there was a reverse correlation between the MED of unprotected skin and the resulting SPF value in the same product. Plotting as a graph the data reported by Damian et al on transmitted dose vs irradiated dose, we observed an exponential‐like behaviour (Figure 2).

**Figure 3:**
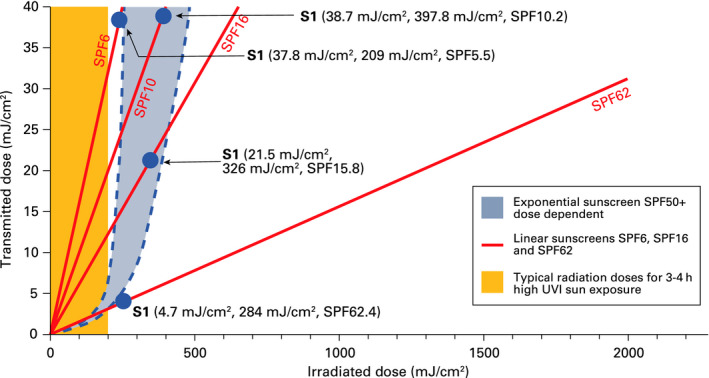
Illustration showing the exponential behaviour of sunscreens and the data for S1 (Table 3)

The exponential sunscreens identified in Table 3 followed a similar exponential‐like behaviour as that reported by Damian et al. In Figure 3, we can see 4 different data points (irradiated dose, transmitted dose) obtained for the sunscreen S1 that follow an exponential pattern in which every point yields a different value of SPF (62, 16, 10 and 6). This raises the question, which of the SPF values is the correct one? According to the purpose of ISO,^2^ it should be the value that best predicts “the protection of human skin from erythema induced by UV radiation from the sun.”

### 3.2 Outdoor clinical study

We next conducted a clinical study in Mauritius during summer time as described in detail under Materials and Methods. In this study, we tested 2 exponential sunscreen products S1 and S7 and compared them to a standard SPF product (P3) and 2 linear sunscreen products (S2 and S3).

The mean clinical erythema scores at 24 hours obtained for the linear sunscreens were S2 (0.3), S3 (0.5) and P3 (1.2) and for the exponential sunscreens were S1 (0.6) and S7 (0.2). Whatever the categorization (linear vs exponential) of the four SPF50+ sunscreens S1, S2, S3 and S7, the mean erythema scores were below grade 1 (equivocal reaction) and there were no statistically significant differences between the exponential products S1 and S7 vs the linear SPF50+ products S2 and S3. There was, however, a statistically significant difference (*P* < .001) when comparing the linear and exponential products to the SPF15 reference standard (P3) and when comparing to the unprotected area**.** In this very high and extreme outdoor UV exposure scenario, the P3 product (SPF15) did not provide sufficient protection from UV radiation.

Interestingly, in the outdoor study, the exponential products S1 and S7, which according to some CROs using high‐dose regimens were predicted to have a very low SPF, performed essentially identically to the linear SPF50+ sunscreens S2 and S3. These results indicate that testing of exponential sunscreen products according to the ISO methodology bears the risk of underestimating in‐use SPF. This suggests that the SPF that best predicts the real protection level in solar exposure conditions is the one determined by CROs working at low dose induced erythema. In the process of SPF testing harmonization, we recommend achieving a consensus on the UV dose regimens used. In addition, if tested under user conditions and with exposure to natural sunlight, differences in sun protection of human skin can no longer be detected between linear and exponential sunscreens. We do not expect that our observations, made in healthy skin, will be different in photosensitive patients.

More studies are required to identify which sunscreen characteristics can help us predict, prior to SPF testing, the category a specific product might fall into. We believe this to be of great clinical relevance because underestimation of the true protection of exponential sunscreens will lead to an increase in the use of UV filters, which would have a negative impact on safety, cosmetic qualities and hence, compliance, with no practical benefits for consumers or the environment.
